# Valsalva maneuver unveils central baroreflex dysfunction with altered blood pressure control in persons with a history of mild traumatic brain injury

**DOI:** 10.1186/s12883-016-0584-5

**Published:** 2016-05-04

**Authors:** Max J. Hilz, Mao Liu, Julia Koehn, Ruihao Wang, Fabian Ammon, Steven R. Flanagan, Katharina M. Hösl

**Affiliations:** Department of Neurology, University of Erlangen-Nuremberg, Schwabachanlage 6, D-91054 Erlangen, Germany; Department of Rehabilitation Medicine, New York University School of Medicine, 240 East 38th Street, New York, NY 10016 USA; Department of Psychiatry and Psychotherapy, Paracelsus Medical University Nuremberg, Prof.-Ernst-Nathan-Strasse 1, 90419 Nuremberg, Germany

**Keywords:** Mild traumatic brain injury, Central autonomic network, Valsalva maneuver, Blood pressure overshoot, Sympathetic dysregulation, Baroreflex dysfunction

## Abstract

**Background:**

Patients with a history of mild TBI (post-mTBI-patients) have an unexplained increase in long-term mortality which might be related to central autonomic dysregulation (CAD). We investigated whether standardized baroreflex-loading, induced by a Valsalva maneuver (VM), unveils CAD in otherwise healthy post-mTBI-patients.

**Methods:**

In 29 healthy persons (31.3 ± 12.2 years; 9 women) and 25 post-mTBI-patients (35.0 ± 13.2 years, 7 women, 4–98 months post-injury), we monitored respiration (RESP), RR-intervals (RRI) and systolic blood pressure (BP) at rest and during three VMs. At rest, we calculated parameters of total autonomic modulation [RRI-coefficient-of-variation (CV), RRI-standard-deviation (RRI-SD), RRI-total-powers], of sympathetic [RRI-low-frequency-powers (LF), BP-LF-powers] and parasympathetic modulation [square-root-of-mean-squared-differences-of-successive-RRIs (RMSSD), RRI-high-frequency-powers (HF)], the index of sympatho-vagal balance (RRI LF/HF-ratios), and baroreflex sensitivity (BRS). We calculated Valsalva-ratios (VR) and times from lowest to highest RRIs after strain (VR-time) as indices of parasympathetic activation, intervals from highest systolic BP-values after strain-release to the time when systolic BP had fallen by 90 % of the differences between peak-phase-IV-BP and baseline-BP (90 %-BP-normalization-times), and velocities of BP-normalization (90 %-BP-normalization-velocities) as indices of sympathetic withdrawal.

We compared patient- and control-parameters before and during VM (Mann-Whitney-U-tests or t-tests; significance: *P* < 0.05).

**Results:**

At rest, RRI-CVs, RRI-SDs, RRI-total-powers, RRI-LF-powers, BP-LF-powers, RRI-RMSSDs, RRI-HF-powers, and BRS were lower in patients than controls. During VMs, 90 %-BP-normalization-times were longer, and 90 %-BP-normalization-velocities were lower in patients than controls (*P* < 0.05).

**Conclusions:**

Reduced autonomic modulation at rest and delayed BP-decrease after VM-induced baroreflex-loading indicate subtle CAD with altered baroreflex adjustment to challenge. More severe autonomic challenge might trigger more prominent cardiovascular dysregulation and thus contribute to increased mortality risk in post-mTBI-patients.

## Background

Traumatic brain injuries (TBIs) are among the leading causes of mortality and disability [1–3]. In the United States, approximately 1.6–3.8 million persons experience a TBI each year [4, 5].

While short-and long-term sequelae are very likely more frequent and intense in patients with a history of moderate or severe TBI than in patients with a history of mild TBI [6, 7], mild TBIs are probably 10 times more frequent than are moderate or severe TBIs [8]. While most patients seem to recover within days or weeks after the mild trauma [9–12], there is increasing evidence of persistent cognitive, psychological, physical and social dysfunction, even after mild TBI [6, 12–15].

In prospective studies of patients with a history of mild TBI, Sung et al. recently showed that depression persists in women for at least 18 months after the trauma [14]. The authors also observed an association between enduring emotional dysfunction [14], lowered levels of insulin-like growth factor [15] and reduced autonomic heart rate modulation after mild TBI. They concluded that altered cardiac autonomic modulation may predict and contribute to enduring emotional disorders after mild TBI [14, 15].

Autonomic dysfunction might even contribute to the long-term increase of mortality rates observed in patients with a history of mild TBI [16–20]. Black and Graham found unremarkable brain autopsies in patients who died unexpectedly but had a history of mild TBI (mTBI) [20]. The authors suggested that dysfunction of central autonomic control might contribute to unexpected fatalities occurring even years after a mTBI [20].

In patients with a history of mTBI (post-mTBI-patients) but without persistent neurological deficits and without any clinically overt autonomic dysfunction, we found an overall decrease in autonomic cardiovascular modulation at rest with a shift towards more sympathetic and less parasympathetic cardiovascular control, and a decrease in baroreflex sensitivity (BRS) [21]. Upon active standing-up, the baroreflex dysfunction became more evident and resulted in less sympathetic activation and less parasympathetic withdrawal than in our healthy control persons [21].

Based on our findings of reduced BRS at rest and subtle impairment of cardiovascular adjustment to baroreflex-unloading upon standing-up, we assume that compromised central BRS modulation may contribute to autonomic cardiovascular dysregulation which in turn might contribute to the increased risk of unexplained fatalities in post-mTBI-patients. Baroreflex mediated changes in sympathetic and parasympathetic activity are induced by impulses that travel via the nucleus tractus solitarii (NTS) towards rostral central autonomic network (CAN) areas [22]. These CAN areas include, e.g., magnocellular neurons of the supraoptic and paraventricular nuclei, posterior hypothalamus, paraventricular and dorsomedial hypothalamic nuclei, preoptic-anterior hypothalamic region, the periaqueductal gray, the central nucleus of the amygdala, and the insular cortex [22].

To further test our hypothesis that post-mTBI-patients have a subtle dysfunction of central baroreflex structures that adjust efferent baroreflex responses, we evaluated in this study whether there is a generalized baroreflex dysregulation in post-mTBI-patients, i.e. whether baroreflex responses are not only altered in response to acute baroreceptor-unloading but also in response to an acute increase in blood pressure (BP) requiring parasympathetic activation and sympathetic withdrawal.

The Valsalva maneuver (VM) is suited to increase BP acutely and non-pharmacologically, and to evaluate baroreflex mediated parasympathetic activation as well as sympathetic withdrawal [22–26]: HR increases during the expiratory strain of a VM, while BP rises to an overshoot after strain-release due to still enduring sympathetic activity [22–25]. The BP-overshoot after strain-release activates the baroreflex and triggers parasympathetic activation with HR-slowing and sympathetic withdrawal with subsequent BP decrease to baseline value [22–26].

In this study, we therefore tested cardiovascular autonomic responses to a VM in patients with a history of mTBI (post-mTBI-patients) but without clinical signs of autonomic dysfunction.

## Methods

In 25 patients (7 women, 18 men, mean age 35.0 years; standard deviation (SD) 13.2 years) who had experienced a mTBI 4 to 98 months prior to examination but had no clinically overt autonomic or other neurological, physical or psychological dysfunction at the time of our autonomic evaluation, we studied HR, BP and autonomic responses to the VM. The interval between autonomic testing and the initial TBI was on average 34 ± 29 months.

Patients were asked to participate in the assessment of autonomic function after we had retrospectively evaluated their medical records, physical and neurological status and TBI severity at the time of the initial trauma.

The initial diagnosis of mild TBI was established by a neurologist or neurosurgeon according to WHO operational criteria including 1) one or more of the following: confusion or disorientation, loss of consciousness for 30 min or less, post-traumatic amnesia for less than 24 h, and/or other transient neurological abnormalities such as focal signs, seizure, and intracranial lesion not requiring surgery; 2) Glasgow Coma Scale (GCS) scores of 13-15 after 30 min post-injury or later upon presentation of health care [27].

We only included patients if they met the above criteria of mild TBI, and if conventional cranial computed tomography (CCT) or magnetic resonance imaging (MRI) showed no abnormality at the time of the initial TBI.

We excluded patients from the study in whom the TBI had occurred due to drugs, alcohol, or medications, to assure that any signs of autonomic dysfunction cannot be related to drugs, medication or other diseases affecting the autonomic nervous system. For the same reason, we also excluded persons with a history of diseases possibly affecting autonomic regulation such as diabetes, cardiac arrhythmias, ischemic heart disease or chronic heart failure, and patients on medication affecting autonomic regulation, e.g., antihypertensive drugs.

Findings in patients were compared to those of 29 age- and sex-matched healthy volunteers (9 women, 20 men, mean age 31.2 ± 12.2 years).

The Institutional Review Board (IRB) of New York University and the ethics committee of the University of Erlangen-Nuremberg, Germany, had approved the study. Written informed consent had been obtained from all participants according to the declaration of Helsinki. All patients reported in this manuscript were recruited at the University of Erlangen-Nuremberg, Germany.

Participants were tested between 9 a.m. and 2 p.m. in a reclining armchair, in a quiet room with 24 °C ambient temperature and stable humidity. Before testing, a resting period of at least 40 min was used to familiarize participants with our equipment and testing procedures, and to assure cardiovascular resting stability.

Using a 3-lead electrocardiogram (ECG), we continuously monitored electrocardiographic RR-intervals (RRI). Systolic and diastolic beat-to-beat BPs (BPsys, BPdia) were recorded continuously at the left hand, using finger pulse photoplethysmography (Portapress; TPD BMI) [25]. In addition, we recorded respiratory frequency (RESP) using a piezoelectric respiratory belt attached to the lower thorax at the point of maximal respiratory excursion [25].

ECG, BP, and respiratory data were continuously sampled, digitized and displayed on a personal computer and a custom designed data acquisition and analysis system (SUEmpathy™, SUESS Medizin-Technik GmbH, Aue, Germany) and stored for off-line analysis.

Cardiovascular autonomic modulation was tested at rest and in response to three reproducible VMs.

To avoid any over-interpretation of potentially abnormal results, we analyzed and interpreted that VM with the values that reflected the least abnormal of the three responses.

### Assessment of time domain parameters of autonomic modulation at rest

From 1-min recordings at rest, we calculated mean values and SDs of all bio-signals. To assess HR variability at rest, we determined the SD and coefficient of variation (CV) of RRIs, both reflecting sympathetic and parasympathetic HR modulation [25, 28], and the square root of mean squared differences of successive RRIs (RMSSD) reflecting parasympathetic influences on HR or RRI variability [25, 28].

### Analysis of spectral powers of autonomic HR- and BP-modulation at rest by Trigonometric Regressive Spectral Analysis (TRS)

HR- or RRI- and BP-values show slow underlying fluctuations that are largely mediated by undulating activity of the autonomic nervous system [25]. Pharmacologic sympathetic or parasympathetic blockade has shown that HR- or RRI- oscillations in the so called low frequency (LF) range from 0.04 Hz – 0.15 Hz at rest are related to sympathetic activity and - to an undetermined degree - also to parasympathetic activity, while LF-oscillations of BP are related to sympathetic outflow only [25, 28]. Moreover, HR- or RRI-oscillations in the so called high frequency (HF) range from 0.15 Hz – 0.5 Hz are associated with respiratory sinus arrhythmia and reflect parasympathetic activity [25, 28], while HF-oscillations of BP-values are primarily a mechanical consequence of respiration-induced fluctuations in venous return and cardiac output [21, 25, 28].

For spectral analysis of slow sympathetically and parasympathetically mediated RRI- and BP-oscillations, we used trigonometric regressive spectral analysis (TRS) of 30 s epochs [21, 29–31]. Spectral analysis algorithms ideally require stationarity of the bio-signal during the epoch of interest [25, 28, 32, 33], a condition that does not exist in biology in strict terms [33, 34] and is moreover not compatible with most algorithms’ requirement of rather long bio-signal time-series recordings [25, 28, 33]. In contrast, the TRS-analysis is suited to evaluate changes in oscillations of bio-signal recordings as short as 25–30 s [29, 30, 35]. The TRS-algorithm thus provides a compromise between the requirement of bio-signal stationarity, the need to assess brief changes in autonomic modulation, and the prerequisite of a bio-signal recording that is still long enough to quantify the slowest of the oscillations of interest [25, 28]. Basically, the TRS-analysis does not depend on the length of the recorded bio-signal time-series but detects physiological signal oscillations within the duration of a sinusoid oscillation of interest [29, 30]. For the estimation of autonomic influences on HR or BP, the slowest sine-waves of interest are in the LF-band, at 0.04 Hz, with a wavelength of 25 s. Thus, the longest recording required for the quantitative analysis of autonomic signal modulation in the LF-band does not exceed 25 s [30] (For further methodological details regarding the TRS-analysis see [21, 29–31]).

We identified peaks of oscillations in the LF- and HF- ranges of RRI-, and BP-modulation [25, 28].

The magnitude of LF- and HF-oscillations was determined as integral under the power spectral density curves of RRI (ms^2^/Hz) and BP (mmHg^2^/Hz) for the two frequency bands, and was expressed as LF- and HF-powers of RRI (ms^2^) and BP (mmHg^2^) [21, 25, 28].

To assess differences in the overall autonomic modulation between the patients and healthy participants, we calculated the sum of LF- and HF-powers as approximation of the total power (TP) of RRI-oscillations and index of overall autonomic cardiac modulation [25, 28]. To determine differences between groups in sympathetic and parasympathetic cardiac modulation, we calculated the ratio between RRI-oscillations in the LF- and HF-ranges as a marker of sympathetic-to-parasympathetic balance [21, 25, 28, 36].

To determine baroreflex sensitivity (BRS), the TRS-software selected pairs of LF- and HF-oscillations of BPsys and RRI with high coherence [37]. Coherence spans from 0, i.e. no association, to 1, i.e. maximum association [10]. High coherence at a specific frequency, e.g., >0.7, indicates a stable phase relation - and thus synchronization - between two signals oscillating at this frequency [10]. Then, the sensitivity of the baroreflex loop (ms•mmHg^-1^) was derived as gain value from changes in RRIs (ms) in relation to changes in BPsys (mmHg) [38].

### The four phases of the Valsalva maneuver

**Fig. 1:**
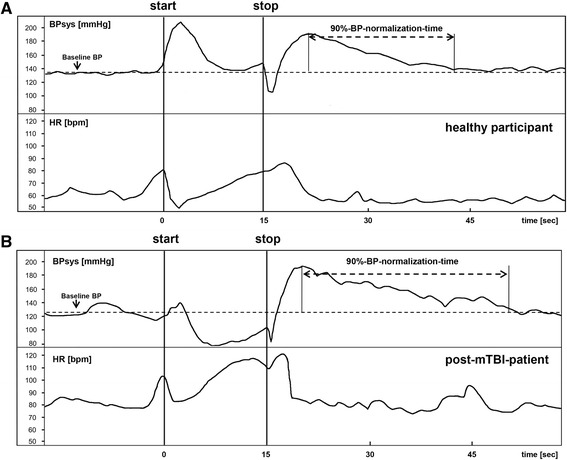
Time-series of systolic blood pressure (BPsys) and heart rate (HR) during the Valsalva maneuver. For a healthy participant and a post-mTBI-patient, the double-headed arrows indicate the intervals from the highest systolic blood pressure (BPsys) value after strain to the time when BPsys had fallen by 90 % of the difference between peak-phase-IV BPsys and baseline BPsys, i.e. the 90 %-blood-pressure-normalization-time. “Start” and “stop” refer to the beginning and release of expiratory strain during the Valsalva maneuver

The VM tests the afferent, central, and efferent sympathetic and parasympathetic baroreflex pathways [39, 40]. VMs were not performed in patients with retinopathy, glaucoma, cerebral aneurysms, dissections or increased intracranial pressure. The VM was standardized by asking the participants to blow into a mouthpiece connected to an aneroid manometer and to maintain a pressure of 40 mmHg for 15 s while the above mentioned bio-signals were continuously recorded [22–25]. The response to the VM includes four phases (Fig. 1). In normal subjects, the sudden increase of intra-thoracic pressure results in a brief rise in BP and in a brief fall of HR (phase I) [22–25]. These changes are mainly the result of mechanical influences. The ongoing strain (phase II) reduces the venous cardiac return which results in a reduction of ventricular dimensions, left ventricular stroke volume and cardiac output. This triggers reflex tachycardia and vasoconstriction [22–25]. The tachycardia during phase II is induced by a prominent early component with inhibited cardiovagal output and a late component with increased sympathetic output [22–25]. In healthy persons, phase II consists of an early fall in arterial BP and subsequent partial recovery. The BP recovery during the late phase II is a result of the progressive increase in total peripheral resistance due to increased sympathetic activity [22–25, 41]. In phase III, a brief fall of arterial BP after the release of intrathoracic pressure reflects mechanical factors [22–25]. The HR shows a reflex increase for usually 3–4 beats [22–25]. The last phase of the normal response to the VM is a rebound overshoot of BP due to the persistent vasoconstriction of the arteriolar bed and to the increased cardiac output that occurs upon release of the forced expiration [22–25]. This BP-overshoot during phase IV activates the baroreflex which results in cardiovagal activation with reflex-bradycardia and in sympathetic withdrawal with peripheral vasodilatation and subsequent decrease in BP [22–25].

During and after the VM, we assessed the maximum of BPsys and BPdia during phases I, II late, and IV, and the minimum of BPsys and BPdia during phases II early, and III, as well as the minimum of RRI during phases II late, and III, and the maximum of RRI during phase IV, within 30 s after strain release.

As index of parasympathetic activation, we calculated the Valsalva-ratio (VR) which is defined as the ratio between the highest HR during and the lowest HR within the first 30 s after the VM, and the time from the lowest to the highest RRIs after strain (VR-time) [22–25]. As index of sympathetic withdrawal, we calculated the interval from the highest BPsys-values after strain-release to the time when BPsys had fallen by 90 % of the differences between peak-phase-IV BPsys and baseline BPsys as 90 %-BP-normalization-time (Fig. 1). Furthermore, we calculated the velocity of BP-normalization, i.e. the difference between the peak-phase-IV BPsys and the BPsys at the moment of the 90 %-BP-normalization-time, divided by the 90 %-BP-normalization-time, and termed this ratio 90 %-BP-normalization-velocity.

### Statistical analysis

We used the Kolmogorov-Smirnov test to test for normal distribution of data. Normally distributed data were presented as mean ± SD. A *t*-test was used to test for significant differences in age between patients and healthy participants. Differences in cardiovascular parameters between patients and healthy participants were evaluated by t-tests for unpaired samples in case of normally distributed data and by the non-parametric Mann-Whitney-U-tests for unpaired samples in case of not normally distributed data. Similarly, we evaluated whether cardiovascular parameters differed between male and female healthy participants as well as male and female post-mTBI-patients. Significance was set at *P* < 0.05. A commercially available statistical program (SPSS™, SPSS Inc., Chicago, Ill, USA) was used for data analysis.

## Results

Age did not differ significantly between post-mTBI-patients (35 ± 13 years) and healthy controls (31 ± 12 years, *P* > 0.05).

At rest, BPsys, BPdia, and RESP did not differ significantly between post-mTBI-patients and healthy controls (Table 1), while RRIs at rest were slightly though not significantly lower in the post-mTBI-patients than in healthy controls.

**Table 1:** Bio-signals and autonomic parameters at rest in 25 post-mTBI-patients and 29 healthy controls

Parameters at rest	Healthy controls	Post-mTBI-patients	Controls vs. patients
RRI [ms]	908.4 ± 132.9	868.7 ± 138.0	*P* = 0.17
BPsys [mmHg]	130.6 ± 17.0	127.5 ± 16.6	*P* = 0.51
BPdia [mmHg]	66.6 ± 8.6	66.1 ± 7.6	*P* = 0.82
Respiration [min^-1^]	13.1 ± 3.8	14.8 ± 3.6	*P* = 0.101
RRI-SD [ms]	59.0 ± 29.3	38.6 ± 18.6	***P = 0.005***
RRI-CV [ms]	6.6 ± 3.3	4.5 ± 2.1	***P = 0.02***
RRI-RMSSD [ms]	48.4 ± 27.8	31.2 ± 18.3	***P = 0.02***
BRS [ms/mmHg]	12.4 ± 5.9	9.2 ± 4.0	***P = 0.043***
RRI-LF-powers [ms^2^]	2417.7 ± 2441.6	953.5 ± 867.8	***P = 0.009***
RRI-HF-powers [ms^2^]	1188.7 ± 1374.6	567.4 ± 581.9	***P = 0.032***
RRI-total-powers [ms^2^]	3606.4 ± 3544.7	1520.9 ± 1300.25	***P = 0.009***
RRI-LF/HF-ratio	3.0 ± 2.1	3.6 ± 3.1	*P* = 0.42
BPsys-LF-powers [mmHg^2^]	12.5 ± 3.9	9.7 ± 4.0	***P = 0.014***
BPsys-HF-powers [mmHg^2^]	5.1 ± 2.2	4.6 ± 3.0	*P* = 0.25

Mean values ± standard deviation of RR-intervals, systolic and diastolic blood pressures, respiratory frequency, time domain parameters of RRI-variability, and frequency domain parameters reflecting powers of autonomic modulation of RR-intervals and systolic blood pressures in 25 patients with a history of mild TBI and 29 healthy controls at rest. Significant differences (*p* < 0.05) between patients with a history of mTBI (post-mTBI-patients) and healthy participants are indicated in italic and bold

*mTBI* mild traumatic brain injury, *RRI* RR-interval, *BPsys* systolic blood pressure, *BPdia* diastolic blood pressure, *SD* standard deviation, *CV* coefficient of variation, *RMSSD* root mean square of the successive differences, *BRS* baroreflex sensitivity, *LF* low frequency, *HF* high frequency

At rest, parasympathetically and sympathetically mediated RRI-SD, RRI-CV, and parasympathetically mediated RMSSD as well as BRS were significantly lower in the post-mTBI-patients than the healthy participants (Table 1).

At rest, RRI-LF-powers, RRI-HF-powers, RRI-total-powers, and BPsys-LF-powers also were significantly lower in the post-mTBI-patients than the healthy participants, while BPsys-HF-powers and RRI-LF/HF-ratios did not differ significantly between both groups (Table 1).

At rest, none of the above parameters differed between healthy men and women or between male and female post-mTBI-patients.

During the VM, patients and healthy participants had similar values of RRI, BPsys, and BPdia in phase I, early phase II, late phase II, phase III, and phase IV, and similar VRs and VR-times (Table 2).

**Table 2:** Bio-signals and autonomic parameters during Valsalva maneuver in 25 post-mTBI-patients and 29 healthy controls

Parameters	Healthy controls	Post-mTBI-patients	Controls vs. patients
RRI phase II late [ms]	643.5 ± 115.4	633.40 ± 85.0	*P* = 0.86
RRI phase III [ms]	628.0 ± 108.5	613.3 ± 78.3	*P* = 0.58
RRI phase IV [ms]	1148.0 ± 160.8	1106.24 ± 208.4	*P* = 0.41
Valsalva-ratio	1.9 ± 0.4	1.8 ± 0.4	*P* = 0.46
Valsalva-ratio-time [s]	13.9 ± 3.8	13.7 ± 3.7	*P* = 0.83
BPsys phase I [mmHg]	166.6 ± 20.6	161.2 ± 13.7	*P* = 0.27
BPsys phase II early [mmHg]	110.0 ± 19.5	105.7 ± 19.8	*P* = 0.44
BPsys phase II late [mmHg]	141.0 ± 23.8	134.0 ± 24.2	*P* = 0.29
BPsys phase III [mmHg]	107.5 ± 17.5	101.1 ± 20.9	*P* = 0.25
BPsys phase IV [mmHg]	177.7 ± 26.6	177.4 ± 21.7	*P* = 0.76
BPdia phase I [mmHg]	88.8 ± 14.3	86.3 ± 11.2	*P* = 0.49
BPdia phase II early [mmHg]	66.8 ± 10.7	67.2 ± 12.44	*P* = 0.90
BPdia phase II late [mmHg]	89.8 ± 13.9	87.0 ± 15.7	*P* = 0.31
BPdia phase III [mmHg]	64.4 ± 9.3	63.9 ± 11.3	*P* = 0.88
BPdia phase IV [mmHg]	85.8 ± 10.5	86.4 ± 10.4	*P* = 0.84
90 %-BP-normalization-time [s]	12.6 ± 4.9	16.9 ± 7.1	***P = 0.01***
90 %-BP-normalization-velocity [mmHg/s]	3.8 ± 1.5	3.2 ± 1.5	***P = 0.04***

Mean values ± standard deviation of maximum or minimum values of RR-intervals, systolic and diastolic blood pressure during the different phases of the Valsalva maneuver, as well as the Valsalva-ratios, Valsalva-ratio-times, 90 %-blood-pressure-normalization-times and 90 %-blood-pressure-normalization-velocities, in 25 patients with a history of mild TBI and 29 healthy controls. Significant differences (*p* < 0.05) between patients with a history of mTBI (post-mTBI-patients) and healthy participants are in italic and bold

*mTBI* mild traumatic brain injury, *RRI* RR-interval, *BPsys* systolic blood pressure, *BPdia* diastolic blood pressure, *BP* blood pressure

**Fig. 2:**
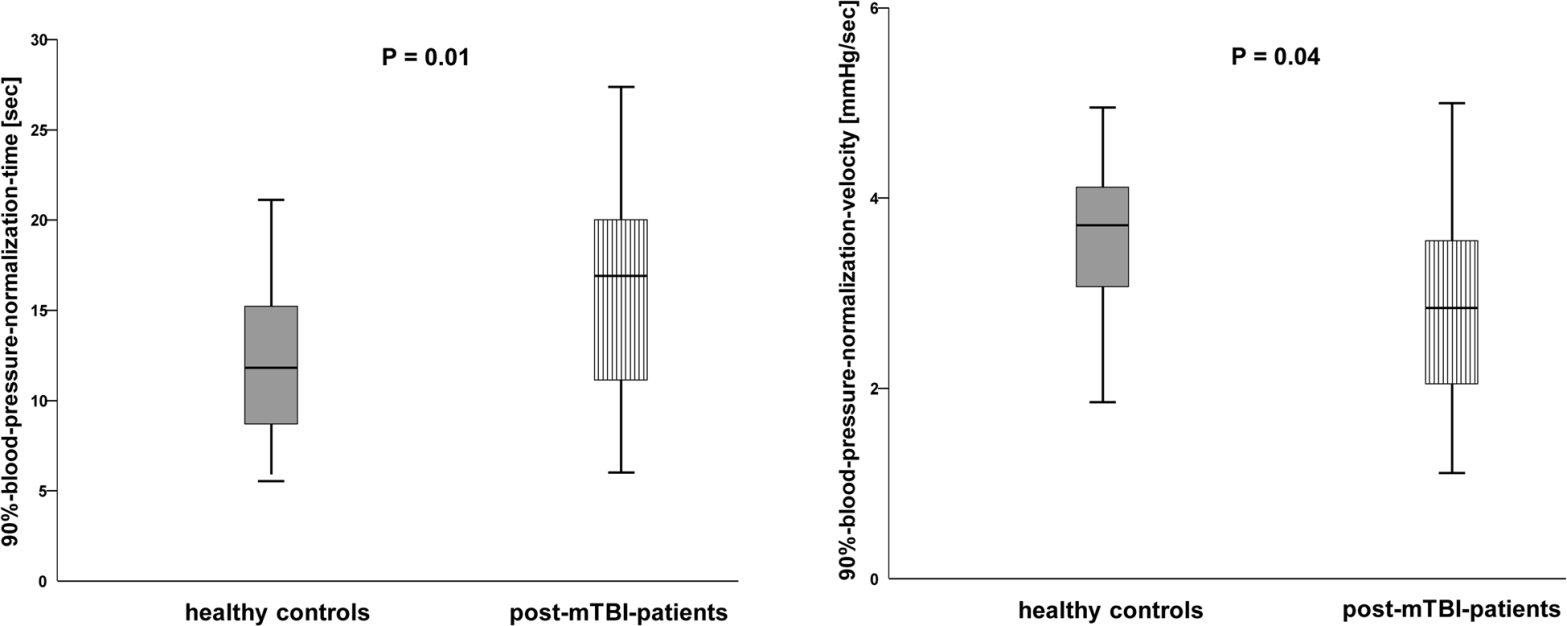
The 90 %-blood-pressure-normalization-times and 90 %-blood-pressure-normalization-velocities in healthy controls and post-mTBI-patients. The 90 % normalization-times of systolic blood pressure (BPsys), i.e. the intervals from the highest BPsys-values after VM strain-release to the times when BPsys had fallen by 90 % of the differences between peak-phase-IV BPsys and baseline BPsys, were longer in the 25 patients with a history of mild TBI than in the healthy participants. The 90 %-blood-pressure-normalization-velocities, i.e. the differences between peak-phase-IV BPsys and BPsys at the moment of the 90 %-BP-normalization-time, divided by the 90 %-BP-normalization-times, were lower in the post-mTBI-patients than in the healthy participants. Data are presented as box plots. The line in the middle of the box represents the median (50th percentile), the top of the box represents the upper quartile (75th percentile), the bottom of the box represents the lower quartile (25th percentile), and the end of the whiskers represent the highest and lowest values that are not extreme values or outliers. Grey boxes illustrate results in healthy participants; white boxes with vertical lines illustrate results in the 25 patients with a history of mild TBI

However, patients had longer 90 %-BP-normalization-times (16.9 ± 7.1 s vs 12.6 ± 4.9 s; *P* = 0.01) than had the controls (Table 2, Fig. 2). Moreover, the 90 %-BP-normalization-velocity was significantly lower in patients (3.2 ± 1.5 mmHg/s) than in controls (3.8 ± 1.5 mmHg/s; *P* = 0.04; Table 2, Fig. 2).

During the VM, only the maximum RRI-values during phase IV, i.e. after strain-release, differed between male and female post-mTBI-patients. Heart rate slowing after strain-release resulted in smaller RRI-values in the female than the male patients (979.0 ± 114.3 ms vs. 1156.9 ± 216.4 ms; *P* = 0.015). Consequently, the VR was lower in the female than the male patients (1.6 ± 0.2 vs. 1.9 ± 0.3; *P* = 0.015), and the VR-time was longer in the female than the male patients (16.2 ± 4.6 s vs. 12.8 ± 2.8 s; *P* = 0.03).

## Discussion

Similar to our previous findings [21], the current data confirm that patients with a history of mTBI have a mild - at rest clinically silent - reduction in the autonomic modulation of HR and BP. Again, all time- and frequency-domain parameters of autonomic HR- and BP- modulation were smaller than in our controls (*P* < 0.05). However, in the current study, RRI-LF/HF-ratios of patients and controls were similar, suggesting that there was no shift towards a predominance of sympathetic over parasympathetic modulation as we had seen it in the post-mTBI-patients of our previous, orthostatic challenge study [21]. Yet, the baroreflex sensitivity at rest was similarly decreased in the patients of the current study as it was in the post-mTBI-patients of the preceding baroreflex-unloading study [21].

Again, the impairment of autonomic modulation seemed to be minor as it did not affect HR- and BP-responses during the various VM-phases. In fact, HR-, BPsys-, and BPdia-values did not differ between patients and controls during the four VM-phases. Not even the Valsalva-ratio, which is commonly determined to assess the cardiovagal baroreflex buffer capacity in response to the BP-overshoot after strain-release [25, 42, 43], nor the times from the lowest to the highest RRIs after strain, the VR-times, differed between our patients and healthy participants.

Only the 90 %-BP-normalization-times and the 90 %-BP-normalization-velocities which we used to measure BP readjustment from maximal BP-values after strain-release to baseline BP-values, and which reflected the quality and velocity of baroreflex mediated sympathetic withdrawal in response to baroreceptor-loading, showed a subtle dysfunction of the central reflex modulation.

This dysfunction seemed to be more prominent in female than in male post-mTBI-patents. In the female patients, heart rate slowing after strain-release was significantly smaller and resulted in smaller RRIs during phase IV of the VM, with subsequently smaller Valsalva ratios and longer VR-times than in the male patients. The findings show that the baroreflex-mediated parasympathetic buffer capacity of heart rate is smaller in female than in male post-mTBI-patients. We cannot rule out that these differences are to some extent due to a slight physiological gender difference in parasympathetic cardiovascular buffering after brief blood pressure increases, as described in previous studies [44, 45]. However, the data might also indicate that persistent autonomic dysfunction is more prominent in women than men after mTBI. The gender related differences show some conformity with recently reported differences between male and female patients regarding autonomic and emotional sequelae of mTBI. Sung et al. found that reduced heart rate variability after mTBI correlated with long-lasting and deteriorating depression in female patients only [14]. Thus, our findings of more severely altered baroreflex function in female than male patients might suggest that women are more prone to persistent autonomic dysfunction after mTBI than are male patients.

Various mechanisms might account for autonomic cardiovascular changes in patients with a history of mild TBI, including the psychological impact of the initial mild TBI, post-traumatic anxiety or depression [12], altered cerebrovascular regulation [13], and particularly prolonged rest after the trauma [13]. Many studies show that physical exercise and endurance training augment cardiovascular autonomic modulation [46–48]; while sedentary life-style and physical inactivity attenuate cardiac autonomic regulation and baroreflex sensitivity [49, 50].

Consequently, the autonomic cardiovascular changes of our patients at rest and after release of the VM-strain, might be ascribed to physical deconditioning after the injury. In fact, various symptoms, including headache, dizziness, fatigue, depression, or cognitive difficulties may persist for an extended period of time in patients even after mild TBI [13, 51]. However, most patients recover swiftly after mild TBI [9, 11, 52, 53]. Similarly, our patients also seemed to have fully recovered from their mild TBI, and none of them had signs or symptoms of clinically overt autonomic dysfunction, psychological or physical impairment, or reported any change in physical activity compared to the level of activity prior to the TBI. Moreover, we tested the patients many months after their TBI; two patients were tested 4 and 5 months after the trauma, four patients were tested after 6 to 12 months, and the remaining 19 patients underwent autonomic testing more than 12 months after the injury. Thus, effects of physical inactivity - which may have occurred during the first months after the TBI – might not explain the decreased autonomic modulation at rest and the delayed withdrawal of baroreflex-mediated sympathetic BP modulation. Ferretti et al. [49] showed that resumption of normal physical activity rapidly reverses attenuating effects of prolonged physical inactivity on autonomic modulation. The authors reported that the decrease in cardiovascular autonomic modulation and BRS induced by 3 months of head-down bed-rest recovered within five days after return to normal activity [49].

In summary, we cannot fully rule out attenuating effects of physical inability or other e.g., psychological impediments, on the cardiovascular autonomic modulation of our patients although these effects seem rather unlikely. Instead, we assume that the autonomic cardiovascular changes at rest and after release of the VM-strain support our previous conclusion that the initial trauma may have caused a minute disruption of CAN pathways even though the trauma was only mild and caused no cerebral lesions visible on conventional CCT or MRI [21]. However, more recent neuroimaging techniques such as diffusion tensor weighted MRI demonstrate microstructural brain lesions even in mild TBI patients suggesting fiber and thus pathway disruption [54–60] or even cerebral volume loss [46] that may persist years after the initial trauma [46, 55, 56, 61].

As mentioned above, efferent responses to loading or unloading of baroreceptors are modulated by many CAN structures distributed throughout the neuraxis [22, 62], e.g., the magnocellular neurons of the supraoptic and paraventricular nuclei, posterior hypothalamus, paraventricular and dorsomedial hypothalamic nuclei, preoptic-anterior hypothalamic region, the periaqueductal gray, the central nucleus of the amygdala, and the insular cortex [22]. While – the assumed - minor lesions within this network did not cause clinically manifest autonomic dysfunction in any of our patients, abundant studies confirm that a decrease in the ability to modulate autonomic function is a risk factor for cardiovascular complications and increased mortality, e.g., in patients with myocardial infarction [63, 64], congestive heart failure [65], renal failure [66], acute ischemic or hemorrhagic stroke [67–70], and diabetes [71–73].

Similarly, the decrease in baroreflex sensitivity, observed already at rest, also indicates an increased cardiovascular risk. Reduced baroreflex function, again, has been shown to correlate with an increased risk of cardiac complications, poor outcome and increased mortality, in various diseases, such as arterial hypertension, heart failure, myocardial infarction, renal failure, diabetes, ischemic or hemorrhagic stroke [63, 74–81].

In a two year prospective study of 1011 community-dwelling 65-year-old persons, Dauphinot and co-workers showed that reduced BRS was associated with the development of arterial hypertension [82]. In a 19-year prospective study comprising 559 middle-aged persons, Kiviniemi et al. recently showed that reduced BRS was the most potent predictor of cardiovascular death [83]. Reduced BRS might even be a predictor of sudden death [75].

The delayed BP decrease after release of the VM-strain further suggests an increased risk of cardiovascular dysregulation. Normally, cardiovascular baroreflex responses to receptor loading or unloading occur almost instantaneously, usually within one heart beat [26]. Any delay in efferent reflex responses may trigger cardiovascular instability [26]. Such delayed cardiovascular baroreflex responses have been reported in various diseases that are associated with an increased cardiovascular risk [84–90].

In our patients, the subtle autonomic changes did not cause any clinically overt abnormality. Still, the finding that our patients needed longer to lower BP after the VM, suggests that they have a mild dysregulation of sympathetic activity. While maneuvers such as our standardized VM or active standing-up only pose a mild autonomic challenge, more pronounced maneuvers might result in more prominent autonomic dysregulation. Maneuvers similar to, but more stressful than a standardized VM are common in daily life, and occur inadvertently, e.g., during lifting heavy weights or even bowel emptying. We assume that a more prominent strain might trigger more severe dysregulation even in patients as ours, with only minor autonomic dysregulation at rest or during mild challenge.

After acute TBI, sympathetic hyperactivity is rather common and has been ascribed to, e.g., axonal injury or hypoxia [91]. In patients with a history of severe TBI, episodes of sympathetic hyperactivity may persist for years [92]. Bursts in sympathetic activity may induce secondary cardiovascular damage including cardiac arrhythmias, myocardial necrosis, cerebral hemorrhage, and even sudden unexplained death [86, 93, 94]. In patients with a history of only mild TBI, such post-concussion sequelae very likely are less frequent and intense than in patients with a history of moderate or severe TBI [95]. Still, daily life activities implying pronounced autonomic cardiovascular challenge might trigger clinically relevant cardiovascular dysfunction even in patients with a history of only mild TBI.

In summary, our study complements and confirms our previous findings of altered baroreflex function upon receptor-unloading [21]. Even patients with a history of only mild TBI may have a dysfunction of autonomic modulation at rest and compromised efferent baroreflex responses upon loading as well as unloading of baroreceptors. Under normal conditions, this dysfunction seems to remain without clinical sequelae. Yet, highly strenuous circumstances or maneuvers might yield an autonomic derangement which could contribute to the increased cardiovascular risk of patients with a history of mTBI [16–20]. In daily life, activities similar to and perhaps even more stressful than a Valsalva maneuver, such as blowing one’s nose or bowel emptying, cannot be avoided and might trigger autonomic dysregulation. Therefore, post-mTBI-patients should be tested for cardiovascular dysregulation upon autonomic challenge, and then closely monitored in an attempt to mitigate the risk of cardiovascular events or emotional and psychiatric complications, as shown e.g. by Sung et al. [14].

## Limitations of the study

Our data reflect the findings in only a rather small number of patients and healthy controls. However, we had to exclude persons from study who were on any medication possibly affecting autonomic function as well as patients who had any clinically overt signs of autonomic dysfunction or any disease afflicting the autonomic nervous system. These exclusion criteria assured that we tested only patients in whom possible findings of autonomic dysfunction could not be ascribed to other causes than the precedent TBI. Still, a larger number of patients and controls would have strengthened the study power and our conclusions.

Another limitation may arise from the wide interval between the times of our autonomic evaluation and the initial mTBI. Patients very likely were at different stages of recovery from mTBI and therefore might have had variable changes in autonomic function. A narrow time frame between the initial trauma and the autonomic assessment might better reflect autonomic function in patients at a more homogenous stage of recovery from mTBI. Yet, Sung et al. recently showed that even changes in heart rate variability recorded within the first week after mTBI are associated with long-term emotional sequelae, such as depression in woman occurring as late as 1.5 years after the mTBI [14]. Our data from patients with a widely dispersed recovery time since their mTBI provide evidence that autonomic cardiovascular dysfunction persists after mTBI not only for several months but for more than 8 years. These findings are in conformity with the studies by Teasdale’s group showing a long-lasting increase in mortality rates, for up to at least 15 years, even after mTBI [18, 19, 96].

Still, the autonomic evaluation of several adequately large patient groups tested after different though narrow intervals since the initial mTBI might show whether and how autonomic dysfunction develops or recovers after mTBI.

Moreover, we cannot rule out that patients who have suffered a mild TBI might tend to have a more stressful life which may increase sympathetic cardiovascular activity. However, in conjunction with our previous studies, our finding of delayed normalization of increased sympathetic activity after the VM challenge suggests a tendency towards increased cardiovascular risk due to an altered sympathetic-parasympathetic balance.

While we cannot prove that the subtle autonomic dysfunction found in our patients causes the increased risk of long-term mortality that has been described in epidemiological studies [16–18, 20], autonomic dysfunction is known to increase the risk of long-term mortality [74–76, 79, 97–99].

## Conclusions

In summary, we conclude that our findings of subtle autonomic and particularly baroreflex dysfunction support our hypothesis that autonomic dysregulation may contribute to cardiovascular imbalance, which in turn increases the risk of cardiovascular events and ultimately death, even years after the initial TBI.

## Ethics (and consent to participate)

The Institutional Review Board (IRB) of New York University, New York, NY, USA, and the Ethics Committee of the University of Erlangen-Nuremberg, Germany, had approved the study. Written informed consent had been obtained from all participants according to the declaration of Helsinki.

## Availability of data and material section

The individual data collected for each participant in our study cannot be provided in order to protect the participants’ identity.
